# Cardiovascular magnetic resonance of scar and ischemia burden early after acute ST elevation and non-ST elevation myocardial infarction

**DOI:** 10.1186/1532-429X-10-47

**Published:** 2008-10-25

**Authors:** Sven Plein, John F Younger, Patrick Sparrow, John P Ridgway, Stephen G Ball, John P Greenwood

**Affiliations:** 1 Academic Unit of Cardiovascular Medicine, University of Leeds, Leeds, UK; 2 Cardiac Magnetic Resonance Unit, Leeds General Infirmary, Leeds, UK; 3 Academic Unit of Medical Physics, University of Leeds, Leeds, UK

## Abstract

**Background:**

The acute coronary syndrome diagnosis includes different classifications of myocardial infarction, which have been shown to differ in their pathology, as well as their early and late prognosis. These differences may relate to the underlying extent of infarction and/or residual myocardial ischemia. The study aim was to compare scar and ischemia mass between acute non-ST elevation myocardial infarction (NSTEMI), ST-elevation MI with Q-wave formation (Q-STEMI) and ST-elevation MI without Q-wave formation (Non-Q STEMI) in-vivo, using cardiovascular magnetic resonance (CMR).

**Methods and results:**

This was a prospective cohort study of twenty five consecutive patients with NSTEMI, 25 patients with thrombolysed Q-STEMI and 25 patients with thrombolysed Non-Q STEMI. Myocardial function (cine imaging), ischemia (adenosine stress first pass myocardial perfusion) and scar (late gadolinium enhancement) were assessed by CMR 2–6 days after presentation and before any invasive revascularisation procedure. All subjects gave written informed consent and ethical committee approval was obtained. Scar mass was highest in Q-STEMI, followed by Non-Q STEMI and NSTEMI (24.1%, 15.2% and 3.8% of LV mass, respectively; p < 0.0001). Ischemia mass showed the reverse trend and was lowest in Q-STEMI, followed by Non-Q STEMI and NSTEMI (6.9%, 14.7% and 19.9% of LV mass, respectively; p = 0.012). The combined mass of scar and ischemia was similar between the three groups (p = 0.17). The ratio of scar to ischemia was 3.5, 1.0 and 0.2 for Q-STEMI, Non-Q STEMI and NSTEMI, respectively.

**Conclusion:**

Prior to revascularisation, the ratio of scar to ischemia differs between NSTEMI, Non-Q STEMI and Q-STEMI, whilst the combined scar and ischemia mass is similar between these three types of MI. These results provide in-vivo confirmation of the diverse pathophysiology of different types of acute myocardial infarction and may explain their divergent early and late prognosis.

## Background

The acute coronary syndromes encompass ST-elevation myocardial infarction (STEMI), non-ST elevation myocardial infarction (NSTEMI) and unstable angina [1,2]. STEMI is typically the consequence of a complete occlusion of the culprit artery with an ultimately fibrin-rich thrombus, whilst NSTEMI is caused by a transient coronary occlusion or of micro-embolisation with components of a non-occlusive, often platelet-rich thrombus [2,3]. As a consequence of these pathophysiological differences, STEMI generally results in larger infarction than NSTEMI [4-6]. Q-waves on an electrocardiogram develop in approximately two thirds of STEMIs, largely dependent on infarct size, but Q-wave development is rare after NSTEMI [7-10].

Whether myocardium supplied by the infarct-related artery remains at risk of further ischemia following acute myocardial infarction (MI) depends largely on the presence of a flow-limiting lesion in the culprit vessel. Furthermore, the amount of viable myocardium remaining at ischemic risk from the culprit lesion is related to the extent of infarcted myocardium; the larger the infarct, the less is left to be at risk of ischemia. Before revascularisation, the combined mass of scar and ischemia represents the total myocardium at risk and should be similar between different types of MI. These basic concepts have not been fully studied in-vivo. In previous studies, scar and ischemia burden after Q-wave and Non-Q wave MI have been compared in segmental models using nuclear scintigraphy [11,12]. Similar comparisons between STEMI and NSTEMI have not been undertaken and quantitative comparisons of scar and ischemia mass are not available.

Cardiovascular magnetic resonance (CMR) offers a potentially more accurate technique for in-vivo comparisons of ischemia, function and scar than nuclear scintigraphy. CMR provides images with high spatial resolution, free of geometric constraints, as well as precise volumetric quantification of abnormalities and direct anatomical correlation [13-15]. In particular, first-pass stress myocardial perfusion and late gadolinium-enhancement imaging offer emerging tools for the in-vivo assessment of coronary heart disease. In this study we used CMR to test the hypothesis that the combined mass of scar and inducible ischemia is similar in NSTEMI, Q-STEMI and Non-Q STEMI reflecting a similar amount of myocardium at risk, but that the ratio of scar to ischemia differs according to the pathophysiology of the infarct.

## Methods

### Subjects

All patients presenting to our institution with a troponin-positive acute coronary syndrome were eligible for study inclusion. Exclusion criteria were previous MI, NYHA class-IV heart failure, ongoing ischemic symptoms, contraindications to CMR or adenosine infusion. All patients gave informed written consent to study protocols approved by our local ethics committee. Patients were prospectively recruited 48-hours after presentation into three predefined groups of NSTEMI, Non-Q STEMI and Q-STEMI. Recruitment into each group was consecutive and unselected until 25 patients were enrolled into each group. The recruitment period was 12 months for NSTEMI, 17 months for Non-Q STEMI and 8 months for the Q-STEMI groups. The groups were defined as follows:

NSTEMI patients had chest pain, no ST elevation on the presenting 12-lead electrocardiogram and elevated troponin levels [16]. According to local protocols patients were initially treated with intensive medical therapy that included aspirin, clopidogrel, heparin and glycoprotein IIb/IIIa inhibitors when indicated. After 2–6 days, they underwent X-ray coronary angiography with the intention to perform revascularisation if required.

Non-Q STEMI patients presented with a first ST-elevation MI according to standard criteria [15]. According to the standard of care at our institution at the time, all STEMI patients were initially treated with intravenous thrombolysis. They underwent X-ray angiography during the index admission only if there was evidence of ischemia [17]. Patients requiring rescue angioplasty were excluded. Patients were recruited to this group if serial electrocardiograms did not show the formation of pathological Q-waves [16].

Q-STEMI patients presented and were managed analogous to the Non-Q STEMI group. They were recruited into this group if they developed pathological Q-waves on serial electrocardiograms over 48 hours [16].

### CMR

All patients underwent CMR between days 2–6 of admission prior to X-ray angiography. CMR studies were carried out on a clinical 1.5 Tesla system (Gyroscan NT Intera CV, Philips Medical Systems, Best, The Netherlands). Heart rate, vectorcardiogram and blood pressure were monitored. The CMR protocol has been described in detail previously [18-21]. It included assessment of LV function, first-pass contrast-enhanced myocardial perfusion at rest and during adenosine-stress as well as late gadolinium-enhanced imaging for the assessment of viability and scar. All data were acquired in LV short axis. First pass myocardial perfusion imaging was carried out at rest and during a five minute adenosine infusion (140 mcg/kg/min). A bolus of 0.05 mmol/kg dimeglumine gadopentetate was given at 6 ml/s by power injector (Spectris, Medrad, Pittsburgh, PA, USA) for each perfusion study and a T1-weighted saturation recovery segmented k-space gradient echo pulse sequence used for data acquisition (repetition time/echo time 3.3/1.6 msec, flip angle 15°, SENSE factor 2, matrix 160 × 112 reconstructed to 256 × 256, spatial resolution 3 × 3 × 8 mm, four slices acquired at each RR interval with a variable interslice gap to cover the LV between apex and base). LV function was assessed with a cine steady state free precession pulse sequence covering the whole LV in 10–12 contiguous slices (repetition time/echo time 2.8/1.4 msec, flip angle 55°, spatial resolution 2 × 2 × 10 mm). Late gadolinium enhancement imaging was performed 10 minutes after the final contrast bolus injection (total dose 0.2 mmol/kg) using an inversion recovery segmented k-space gradient echo pulse sequence (repetition time/echo time 7.5/3.8 msec, flip angle 15°, identical geometry to LV cine images, spatial resolution 1.3 × 1.3 × 10 mm, inversion time set to null signal from normal myocardium).

Perfusion imaging in this study covered the LV in four short axis slices with a variable interslice gap, whilst late gadolinium-enhanced imaging provided LV coverage in 10–12 slices with no interslice gap. This approach was necessary because with current CMR technology the number of slices that can be acquired in first pass perfusion studies is limited unless sacrifices in temporal resolution, in-plane spatial resolution or other determinants of image quality are made. For this study we regarded in-plane spatial resolution and good image quality as more relevant than acquiring additional slices in the perfusion studies. In order to establish the potential bias introduced by comparing the full-coverage late gadolinium-enhanced imaging with the four-slice perfusion assessment, 10 randomly selected late gadolinium-enhanced data sets were analysed further. The data were reanalysed using only 4 slices with a 10 mm interslice gap (as would be typical for a perfusion study) and following interpolation, absolute LV mass, scar mass and percentage scar mass calculated. Compared with the full-coverage data sets, no significant differences were seen in percentage scar mass of the decimated data sets (mean error: 0.4% p = 0.42).

### CMR analysis

Analysis was performed using Mass 5.0 software (Medis, Leiden, The Netherlands). On the cine images LV endocardial and epicardial borders were outlined in diastole and systole. Ischemia was defined visually as reduced or delayed contrast uptake on stress perfusion images in myocardium outside of the scar zone (on corresponding late gadolinium enhancement images). Ischemic myocardium in the infarct related artery territory only, as well as LV endocardial and epicardial borders were outlined manually with separate contours. On late gadolinium-enhanced images, areas of enhanced myocardium (signal intensity more than 2SD above normal myocardium) were outlined as previously described [15]. Finally, images were segmented according to the America Heart Association classification [22].

Using manual planimetry and summation of discs methodology, LV volumes, ejection fraction and LV mass were calculated from the cine images covering the whole heart. Ischemia mass and the LV myocardial mass covered by the perfusion images were calculated. Likewise, scar and LV myocardial mass covered by the late gadolinium enhancement images were computed. Values were expressed as absolute mass (g) and as percentage of LV mass relative to the total myocardium covered by the respective acquisition. The ratio of ischemia to scar was calculated by dividing percentage ischemia by percentage scar.

### X-ray angiography

Cardiac catheterization was carried out using a standard clinical technique and was reported by a blinded interventional cardiologist. The presence of one or more coronary stenoses of >70% luminal narrowing in a main coronary vessel or major side branch of >2 mm diameter was reported as significant. The 'culprit lesion' was defined on the basis of angiographic characteristics, regional wall motion abnormality and location of ECG changes.

### Statistical analysis

Continuous variables are presented as mean ± SD, and compared using one way analysis of variance with Bonferroni multiple post test comparisons. Categorical data are presented as number (%) and compared using a chi-square test. The least square technique was used to assess the linear relationship between variables. All statistical tests were 2-sided and performed at the 5% significance level.

## Results

### Clinical characteristics

The study group consisted of 75 patients; 25 recruited consecutively into the three groups of NSTEMI, Non-Q STEMI, Q-STEMI; demographics are listed in Table 1. There were no significant differences in age, gender, risk factors or presenting characteristics in multiple comparisons between the three groups.

**Table 1:** Demographics and presenting characteristics of patients in the three study groups.

	NSTEMI (n = 25)	Non-Q STEMI (n = 25)	Q-STEMI (n = 25)
Age – yr	57 (± 9.3)	60 (± 9.1)	57 (± 8.9)
Male	22 (88%)	20 (80%)	23 (92%)

**Risk factors – no. (%)**			

Diabetes mellitus	5 (20%)	1 (4%)	6 (24%)
Hypertension	9 (36%)	3 (12%)	4 (16%)
Known CAD	3 (12%)	2 (8%)	2 (8%)
Family history of CAD	13 (52%)	8 (32%)	10 (40%)
Current smoker	12 (48%)	12 (48%)	15 (60%)
Previous revascularisation	0	0	0

**Presenting characteristics**			

Systolic BP (mmHg)	129 (± 17.1)	133 (± 21.7)	128 (± 18.8)
TIMI score*	2.7 (± 1.4)	2.1 (± 1.4)	2.7 (± 2.1)

*The TIMI risk scores for NSTEMI and STEMI are different, so that the only statistical comparison is between Non-Q STEMI and Q-STEMI (unpaired t-test). CAD = coronary heart disease; BP = blood pressure.

The time between symptom onset and thrombolysis was 244 ± 208 min in the Q-STEMI and 168 ± 157 min in the Non-Q STEMI group (p = 0.15). Biomarkers of myocardial damage were significantly lower in the NSTEMI compared with the Non-Q STEMI and Q-STEMI groups (troponin-I 5.9, 29.6 and 69.9 mg/ml, respectively, p < 0.0001; peak CK 396, 1289, 2253IU, respectively, p < 0.0001) (Table 2). Infarct location was anterior in 12 and inferior/lateral in 13 of Q-STEMIs and anterior in 8 and inferior/lateral in 17 of Non-Q STEMIs.

**Table 2:** Biomarkers, LV volumes, LV mass, ischemia and scar burden in the three study groups.

	A NSTEMI (n = 25)	B Non-Q STEMI (n = 25)	C Q-STEMI (n = 25)	A vs B p*	A vs C p*	B vs C p*
Troponin I (mg/ml)	5.9 (± 15.7)	29.6 (± 30.3)	69.9 (± 60.4)	>0.05	<0.001	<0.05
Peak CK (IU)	396 (± 421.8)	1289 (± 818)	2253 (± 1524)	<0.05	<0.001	<0.01
Anterior MI	-	8	12			
Inferior/lateral MI	-	17	13			

LVEDV (ml)	172.5 (± 51.3)	175.0 (± 40.8)	186.9 (± 29.3)	>0.05	>0.05	>0.05
LVESV (ml)	80.0 (± 44.8)	94.9 (± 32.5)	107.1 (± 25.1)	>0.05	<0.05	>0.05
LV EF (%)	55.5 (± 9.5)	46.6 (± 7.3)	43.1 (± 7.8)	<0.001	<0.001	>0.05
LV mass (g)	133.1 (± 36.3)	115.3 (± 26.7)	131.7 (± 24.9)	>0.05	>0.05	>0.05

Scar %	3.8 (± 5.3)	15.2 (± 8.4)	24.1 (± 12.6)	<0.001	<0.001	<0.01
Ischemia %	19.9 (± 20.1)	14.7 (± 12.6)	6.9 (± 11.4)	>0.05	<0.01	>0.05

Data presented as mean ± SD.

* All statistical comparisons by ANOVA post hoc tests. CK = creatine kinase; LV = left ventricle; EDV = end diastolic volume; ESV = end systolic volume; EF = ejection fraction.

X-ray coronary angiography was carried out in all patients in the NSTEMI group, 19 in the Non-Q STEMI and 18 in the Q-STEMI group. Table 3 shows the disease distribution and culprit lesion location. Three patients in both STEMI groups had persistently occluded culprit vessels. By chi-square analysis, there were no significant differences between the three groups (NSTEMI, Non-Q STEMI, Q-STEMI) in terms of their angiographic distribution of vessels with significant stenosis, location of the culprit lesion, or the disease extent (single, two-vessel or three-vessel disease).

**Table 3:** X-ray angiographic results of patients in the three study groups.

	NSTEMI (n = 25)	Non-Q STEMI (n = 19)	Q-STEMI (n = 18)
**Vessels with stenosis >70%**			

LMS	1 (4%)	0	0
LAD	9 (36%)	8 (42%)	10 (55%)
Cx	10 (40%)	9 (47%)	6 (33%)
RCA	9 (36%)	6 (32%)	5 (28%)

**Culprit lesion**			

LMS	1 (4%)	0	0
LAD	9 (36%)	7(37%)	10 (55%)
Cx	5 (20%)	8(42%)	4 (21%)
RCA	5 (20%)	4(21%)	4 (21%)

**Disease extent**			

Minor atheroma only	5	4	3
Single vessel	14 (56%)	8 (42%)	9 (50%)
Two-vessel	4 (16%)	6 (31%)	6 (33%)
Three vessel	2 (8%)	1 (5%)	0

LMS = left main stem; LAD = left anterior descending artery; Cx = circumflex artery; RCA = right coronary artery.

### CMR

No events occurred between recruitment and CMR, and all 75 CMR studies were completed. The results for LV function parameters are listed in Table 2. Total LV mass and end diastolic volumes were similar between all three groups (overall and post hoc comparisons = NS). End systolic volumes were significantly different between the 3 groups (p = 0.029), with higher volumes in the Q-STEMI vs. NSTEMI groups (p < 0.05). LV ejection fraction was also significantly different between the 3 groups (p < 0.0001), with greater values in the NSTEMI group vs. both Non-Q STEMI (p < 0.001) and Q-STEMI (p < 0.001).

**Figure 1:**
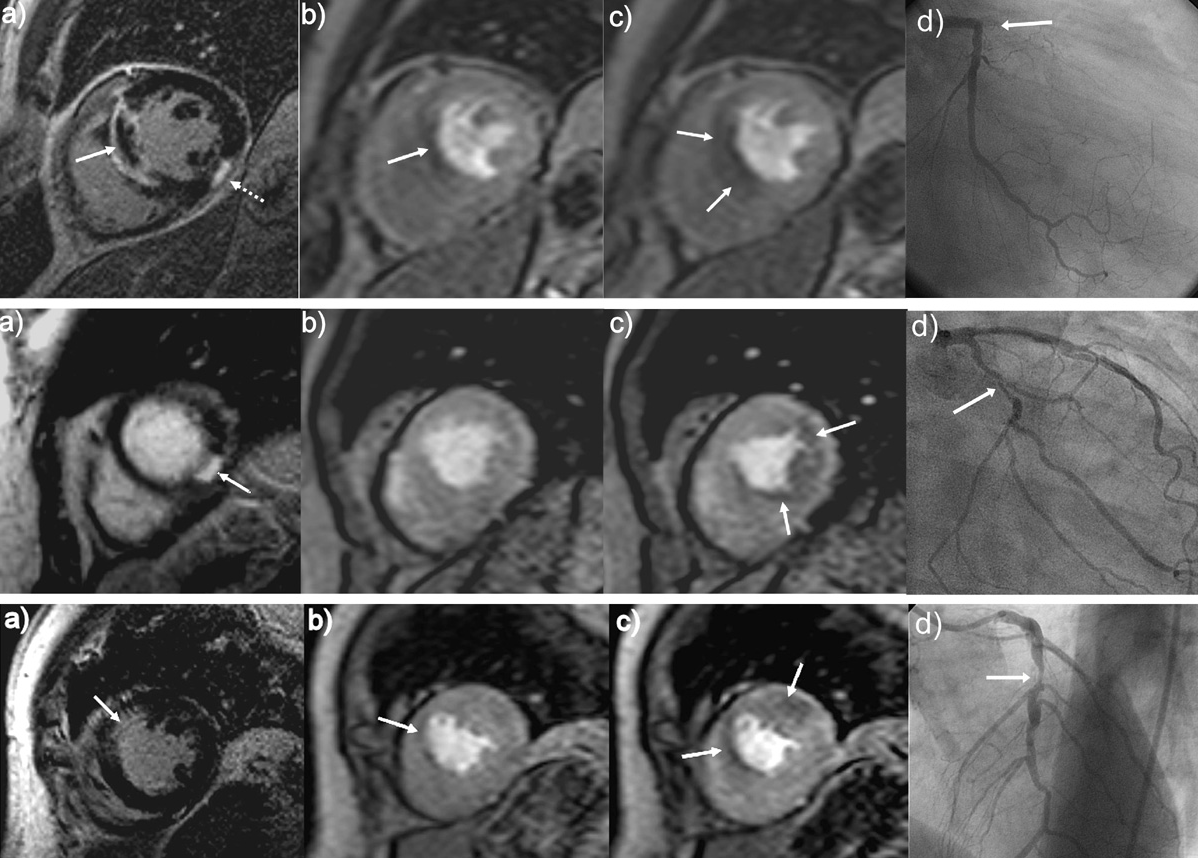
***Column A *= Late gadolinium-enhancement CMR in the short axis.***Column B *= Rest perfusion CMR at peak myocardial enhancement in the identical location. *Column C *= Adenosine stress perfusion CMR with identical image parameters to column B. *Column D *= Corresponding X-ray coronary angiogram. *Top Row *= Example of Q-wave STEMI, showing a large septal scar with a central area of microvascular obstruction (A; full arrow). There is also a small inferior scar (A; dotted arrow). Rest perfusion CMR (B) shows a defect corresponding predominantly to the area of the microvascular obstruction. Stress perfusion CMR (C) shows the perfusion defect in the entire infarct and extending marginally into the peri-infarct zone. Coronary angiography (D) revealed an occluded proximal LAD at the site of a previous stent. *Middle Row *= Example of Non Q-wave STEMI, showing a small inferior scar (A; arrow). Rest perfusion CMR (B) shows the small inferior scar is not detected as a fixed perfusion defect. The stress perfusion image (C) shows a large inducible perfusion defect infero-laterally, extending beyond the scar into the peri-infarct zone (C; arrow). The coronary angiogram (D) shows a severe stenosis in the mid circumflex artery. *Bottom Row *= Example of NSTEMI, showing a small subendocardial scar in the antero-septal segment (A). Rest perfusion CMR (B) appears homogenous outside the scar. The stress perfusion image (C) shows a large area of inducible antero-septal ischemia. On coronary angiography (D), severe disease in the left anterior descending artery was found.

Example images of study patients with NSTEMI, Non-Q STEMI and Q-STEMI are given in Figure 1. They illustrate the different extent of scar and ischemia in the three patient groups.

### Scar burden

All patients with Q-STEMI and Non-Q STEMI had evidence of myocardial scar on late gadolinium-enhancement images, whilst 7 (28%) NSTEMI patients showed no focal scar. The scar burden expressed as percentage of LV mass was significantly different between the 3 groups (p < 0.0001), and was largest in Q-STEMI, followed by Non-Q STEMI and NSTEMI (Table 2). In 20 (80%) of the patients with Q-STEMI and 20 (80%) of the Non-Q STEMI patients the scar was more than 75% in transmural extent in at least one segment, whilst only one (4%) patient with NSTEMI showed transmural scar.

### Ischemia and total myocardium at risk

Ten (40%) patients with Q-STEMI, 19 (76%) patients with Non-Q STEMI and 15 (60%) patients with NSTEMI had evidence of inducible ischemia on stress-perfusion CMR (p = 0.03). The volume of ischemia was lowest in Q-STEMI, followed by Non-Q STEMI and NSTEMI (6.9%, 14.7% and 19.9% of LV mass, respectively; p = 0.012); Table 2.

**Figure 2:**
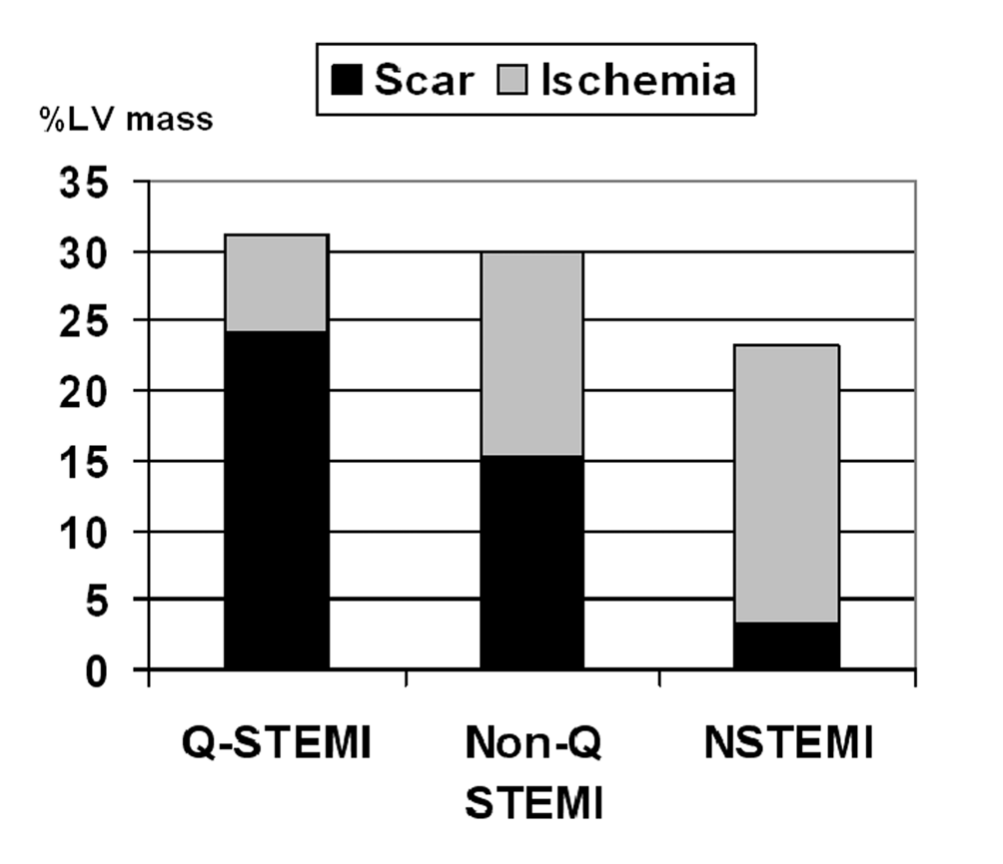
**Scar and ischemia burden**. Scar and inducible ischemia burden in Q-STEMI, Non-Q STEMI and NSTEMI expressed as percentage of LV mass.

Scar and ischemia burden combined accounted for 31% of total LV mass in Q-STEMI, 29.9% for Non-Q STEMI and 23.1% for NSTEMI, (p = 0.17); (Figure 2). The ratio of scar to ischemia was 3.5, 1.0 and 0.2 for Q-STEMI, Non-Q STEMI and NSTEMI, respectively.

### Correlation analyses

Peak CK and percent LV infarct mass were significantly positively correlated (r = 0.85, p < 0.0001). Troponin-I and percent LV infarct mass were less strongly correlated (r = 0.65, p < 0.0001). For ejection fraction and percent LV infarct mass there was a significant negative correlation (r = -0.65, p < 0.0001).

## Discussion

This study provides in-vivo confirmation of the pathophysiological differences between different types of acute myocardial infarction. The data show that the ratio of scar to ischemia varies significantly, with STEMI leading to larger infarcts than NSTEMI, and that Q-STEMI is associated with a higher scar burden than Non-Q STEMI. Conversely, ischemia burden is lower after Q-STEMI than in both Non-Q STEMI and NSTEMI. Combined however, the total mass of scar and ischemic myocardium at risk is similar between all three types of infarction.

Unlike previous studies that have relied primarily on biomarkers to determine infarct size or nuclear scintigraphy with its limited spatial resolution, measurements were derived using high resolution CMR. Importantly, because of the clinical protocols at the time of the study, we were able to obtain imaging data prior to any revascularisation procedures so that residual inducible ischemia in the infarct-related artery territory could be determined. Increasingly, patients with STEMI undergo primary angioplasty and NSTEMI patients are rapidly referred for angiography-guided revascularisation, so that comparable studies to this will be difficult to conduct in the future.

### STEMI versus NSTEMI

The main finding from this study was that the combined scar and ischemic myocardial mass was similar between patients with STEMI and NSTEMI, and only the ratio of ischemia versus scar differed. While previous separate studies in STEMI and NSTEMI have shown larger infarct size in STEMI [4-6], the current study provides the first direct in-vivo comparison of infarct size between these two sub-types of acute MI. In our population, patients with STEMI had more transmural scars and on average a significantly larger infarct mass than patients with NSTEMI. Ejection fraction inversely mirrored the scar mass and was significantly lower early after STEMI than after NSTEMI. These observations confirm the theory that in the absence of adequate collateral supply, myocardial necrosis expands for as long as the coronary occlusion persists. The occlusive thrombus responsible for STEMI therefore causes larger infarcts than the transient coronary occlusion or distal embolisation responsible for NSTEMI.

Ischemia burden on the other hand was both more common and more extensive in the NSTEMI group than in either of the STEMI groups. This finding is biologically plausible as the total perfusion zone of the infarct related arteries will be similar between STEMI and NSTEMI. Because in STEMI more myocardium is infarcted, less tissue is left at ischemic risk after the acute event.

### Q-STEMI versus Non-Q STEMI

In approximately one third of patients suffering a STEMI pathological Q-waves do not develop on the post-infarct electrocardiogram [7,8]. It is now widely accepted that the development of Q-waves is mainly a reflection of infarct size [9,10]. Potential pathophysiologic mechanisms for the smaller infarct size in Non-Q STEMI include a shorter duration of ischemia, a lower thrombus burden and more distal stenosis. In our study, patients with Non-Q STEMI formed an 'intermediate' group compared to Q-STEMI and NSTEMI in terms of infarct size, LV function and residual inducible ischemia. The presenting characteristics, infarct location and angiographic features were similar in Q-STEMI and Non-Q STEMI patients. Only time to treatment was shorter in Non-Q STEMIs, although this difference did not reach statistical significance and no difference in time to treatment was seen in previous larger studies [8]. Our results are in accord with prior observations that scar size is smaller in Non-Q STEMI, ejection fraction is higher and cardiac enzyme release is less than in Q-STEMI [7,8].

Previously, Q-wave and Non-Q-wave MI have been compared by nuclear scintigraphy methods. As long as 20 years ago, Gibons *et al*., reported less segments with persistent 201Tl defects and more segments with redistribution defects during stress in Non-Q-wave MI than Q-wave MI [11]. More recently, Yang *et al*., reported higher ischemia burden in Non-Q-wave than Q-wave MI using positron emission tomography, but in contradiction to both our study and Gibons' results, they observed no significant difference in scar burden between the two types of MI [12]. Whilst differences in patient selection between our study and Yang's study may be partly responsible for these discrepant results, importantly, in our study the high spatial resolution of CMR allowed scar and ischemia to be measured in absolute grams of tissue rather than by myocardial segments as is custom in nuclear scintigraphy. Our results should thus give a more accurate reflection of the pathological effects of different types of acute infarction than the previous literature.

### Limitations

Stress perfusion imaging will only reveal ischemia in those patients with an underlying flow-limiting coronary stenosis. It is well recognized that a significant proportion of acute coronary syndromes arise from plaques that are not flow-limiting [2]. The true prevalence of flow-limiting disease in our population is not fully known because not all STEMI patients underwent coronary angiography and invasive pressure wire measurements were not performed. In those patients who had angiography however, approximately 20% in all three study groups had no coronary stenosis of greater than 70% severity.

The stress perfusion sequence did not allow full LV coverage. Even after correcting for this with data interpolation, there is still the possibility that inducible ischaemia could have been missed from a true basal or apical slice. This would likely have impacted most on the NSTEMI group where the ischaemic burden was greatest; indeed the combined scar and inducible ischaemia mass in this group was less (Figure 2) than the other two groups.

Finally, there will always be individual variation as to the mass of myocardium supplied by a particular coronary artery and also the size of the 'territory at risk' depending on the location of acute lesion. Although this can never be strictly controlled for in human studies, there were no significant differences in angiographic characteristics between our three groups.

### Clinical implications

In STEMI the risk of adverse events is considered highest during the acute presentation, likely related to the larger scar mass and lower ejection fraction, whilst relatively less myocardium remains at long-term ischemic risk. However in patients with NSTEMI, despite an initial lower risk, cardiovascular events in the longer term are at least as frequent, if not more so, compared to STEMI [23-28]. The results of this study support the notion that patients with NSTEMI lose their initial prognostic advantage, as more viable tissue in the territory of the infarct-related artery remains at ischemic risk [14]. Even though a proportion of culprit lesions are not flow-limiting, inducible ischemia is more common after NSTEMI and than after Q-STEMI. Early revascularisation can thus improve prognosis after NSTEMI by removing the substrate for subsequent ischemic events, and is recommended in current management guidelines [29].

## Conclusion

This study has demonstrated that clear differences exist in the mass of scar and ischemic myocardium at risk between Q-STEMI, Non-Q STEMI and NSTEMI, whilst overall the combined mass of ischemic and scar tissue was similar. The greater mass of inducible ischemic tissue in NSTEMI patients may be one of the factors responsible for their high late cardiovascular event rate, if appropriate revascularisation is not undertaken.

## Competing interests

The authors declare that they have no competing interests.

## Authors' contributions

All authors were responsible for study design, conduct and analysis, and all read and approved the final manuscript.
